# Transcriptional Response of Human Neurospheres to Helper-Dependent CAV-2 Vectors Involves the Modulation of DNA Damage Response, Microtubule and Centromere Gene Groups

**DOI:** 10.1371/journal.pone.0133607

**Published:** 2015-07-24

**Authors:** Stefania Piersanti, Romina Burla, Valerio Licursi, Catarina Brito, Mattia La Torre, Paula M. Alves, Daniel Simao, Carla Mottini, Sara Salinas, Rodolfo Negri, Enrico Tagliafico, Eric J. Kremer, Isabella Saggio

**Affiliations:** 1 Department of Biology and Biotechnology “C. Darwin”, Sapienza University of Rome, Rome, Italy; 2 Pasteur Institute, Cenci Bolognetti Foundation, Rome, Italy; 3 iBET, Instituto de Biologia Experimental e Tecnológica, Apartado 12, 2780–901, Oeiras, Portugal; 4 Instituto de Tecnologia Química e Biológica, Universidade Nova de Lisboa, Av. da República, 2780–157, Oeiras, Portugal; 5 Department of Biomedical Sciences, University of Modena and Reggio Emilia, Modena, Italy; 6 Institut de Génétique Moléculaire de Montpellier, CNRS UMR 5535, Montpellier, France; 7 Université de Montpellier, Montpellier, France; 8 Institute of Molecular Biology and Pathology, CNR, Rome, Italy; Swedish Neuroscience Institute, UNITED STATES

## Abstract

Brain gene transfer using viral vectors will likely become a therapeutic option for several disorders. Helper-dependent (HD) canine adenovirus type 2 vectors (CAV-2) are well suited for this goal. These vectors are poorly immunogenic, efficiently transduce neurons, are retrogradely transported to afferent structures in the brain and lead to long-term transgene expression. CAV-2 vectors are being exploited to unravel behavior, cognition, neural networks, axonal transport and therapy for orphan diseases. With the goal of better understanding and characterizing HD-CAV-2 for brain therapy, we analyzed the transcriptomic modulation induced by HD-CAV-2 in human differentiated neurospheres derived from midbrain progenitors. This 3D model system mimics several aspects of the dynamic nature of human brain. We found that differentiated neurospheres are readily transduced by HD-CAV-2 and that transduction generates two main transcriptional responses: a DNA damage response and alteration of centromeric and microtubule probes. Future investigations on the biochemistry of processes highlighted by probe modulations will help defining the implication of HD-CAV-2 and CAR receptor binding in enchaining these functional pathways. We suggest here that the modulation of DNA damage genes is related to viral DNA, while the alteration of centromeric and microtubule probes is possibly enchained by the interaction of the HD-CAV-2 fibre with CAR.

## Introduction

Due to the structural and functional compartmentalization of the central nervous system (CNS), some brain pathologies can be challenging to understand and treat. A powerful therapeutic approach is the use of virus-based gene transfer vectors that allow long-term gene expression [1,2]. In this scenario, helper-dependent (HD) canine adenovirus type 2 (CAV-2) vectors are promising tools. Wild type CAV-2 does not propagate in humans and therefore clinical use of these vectors should not be markedly limited by pre-existing immunity. Importantly, CAV-2 vectors preferentially transduce neurons *ex vivo* in human organotypic brain slices, and mouse, rat and dog neurons *in vitro* and *in vivo*. They also efficiently traffic via retrograde axonal transport to afferent structures [3]. In addition, HD-CAV-2 vectors are poorly immunogenic in the CNS [4,5], and can harbor a variety of expression cassettes [6–8].

While the intrinsic characteristics of HD-CAV-2 vectors make them applicable for the therapy of some neurodegenerative disorders, a more complete understanding of the biological events occurring during the interaction between HD-CAV-2 and neurons is a prerequisite for their efficient use. A second aspect prompting to analyze the effect of CAV-2 on the host cell is that it can help address key neurobiological questions. Studies on CAV-2 interaction with the coxsackievirus and adenovirus receptor (CAR), the mediator of CAV-2 attachment and entry into the neuron, have provided insight into CAR biology. CAR is a widely expressed cell adhesion protein belonging to the Ig superfamily [9]. Although CAR can function as a cell adhesion molecule in epithelial cells, its role is poorly defined in neurons. Studies with CAV-2 have shown that, upon interaction with CAV-2 or with CAV-2 fibre knob (FK^CAV^), CAV-2 and CAR can be co-transported in axons or induce CAR degradation [3,10,11]. Together these studies suggested for a role of CAR in neuron homeostasis, adhesion and axonal transport. Independent experiments based on the use of human adenovirus type 5 (HAd5) established a link between HAd5, CAR, microtubules and cell migration [12], and between CAR and neurite extension [13]. Interestingly, a virus-independent interaction of CAR with microtubules was seen by biochemical experiments showing a direct interaction [14].

One approach to define the toxicogenomic signature of vectors and to characterize the biological pathways perturbed by vectors is to perform a genome wide transcriptome analysis of the transduced target cells. Global transcriptional analysis has been applied to the study of HAd5, HIV-1 (LV) and AAV vectors [15–21]. We previously used genechips to dissect and compare the response to HD-HAd5 to that of E1-deleted HAd5 vectors in hepatic cells, finding that E1-deleted and HD-HAd5 induce a response of equal magnitude, but with different properties [19]. Di Pasquale et al. assayed AAV5-based vectors and reported the PDGF receptor as a functional AAV2 receptor [15]. In a recent study, we compared the toxicogenomic signature of bidimensional (2D) cultures of human midbrain derived neuronal progenitor cells (hmNPCs) transduced with HD-CAV-2, LV and HD-HAd vector [22,23]. Through these analyses we concluded that the DNA damage and cell cycle regulation responses were affected by HD-CAV-2 transduction in the 2D system, which was vector dependent and vector specific. Doronin and co-workers used genechips to establish a link between coagulation factor X (FX), a co-receptor of human Ad, and the activation of the innate response in vivo by group C HAd [24].

Here we analyzed the response of 3D cultures of human neural cells to HD-CAV-2. The use of neurospheres is of interest for the understanding of the molecular and cellular pathways implicated in neurological disorders. Neurospheres are particularly suited for preclinical studies because they mimic several aspects of the human brain. Notably, our recent study described a differentiation protocol applied to 3D cultures of hmNPCs that allow the production of tissue-like structures containing functional dopaminergic neurons undergoing synaptogenesis and spontaneous Ca^2+^ transients, and recapitulating midbrain neuron patterning events [25,26].

Herein we report the transcriptional signature induced by HD-CAV-2 in this *bona fide* brain cell model. We demonstrated that HD-CAV-2 permits high transduction efficiency, and provokes the modulation of probes functionally belonging to the DNA damage pathway and to the centromeric and microtubule metabolism.

## Materials and Methods

### Vectors and cells

HD-HAd was produced as described previously [27]. LV was prepared by combined transfection of 293T cells with the following plasmids: pRRLSIN.cPPT.PGK-GFP.WPRE, pMDLg/pRRE, pRSV-Rev and pMD2G (all from Addgene, http://www.addgene.org/). Cell supernatants were collected at 48 and 72h and successively purified as described [28]. HD-CAV-2 was produced as previously described [4]. Titers of HD-HAd, LV, and HD-CAV-2 were determined by Q-PCR on vector genomes as previously described [22].

Human midbrain-derived neural progenitor cells (hmNPCs) derived from aborted fetal brain tissue 12 to 14 weeks post-fertilization were provided by Dr. Johannes Schwarz (Technical University of Munich, Germany) in the context of the EU FP7 BrainCAV (n. 222992) grant agreement. Tissue was obtained with written mother’s consent and in accordance with the Ethics Committee of the University of Leipzig and the German state and federal laws. Two independent hmNCPs batches (BNA and 3821, as per indications of Johannes Schwarz) were cultured and differentiated. Unless specifically indicated BNA cells were used. Expansion of hmNPCs was performed on poly-L-ornithine-fibronectin (PLOF)-coated surfaces and serum-free medium, as described previously [29–31]. 2D cultures of differentiated hmNPCs were prepared as described previously [22]. To produce neurospheres, hmNPCs were cultured in dynamic culture systems as previously described [31]. 2D cultures were transduced with different viral vectors at an MOI of 1000 vg/cell as previously described [22]. 3D cultures of hmNPCs were similarly transduced at an MOI of 1000 vg/cell, for 2 h [22]. CAV-2 fibre knob (FK^CAV^) (residues 358–542) was prepared as described [32] and added to cells at a concentration of 2.5 μg/1.5 x 10^6^ cells for 2 h. Next, cells were washed and total RNAs collected at 5 days.

### Microscopy

GFP was analyzed on cells fixed with 4% paraformaldehyde and 2% sucrose. Cells were mounted in DAPI-Vectashield (Vector laboratories) to stain DNA. Slides were analyzed with a Zeiss Axioplan epifluorescence microscope and with a Leica TCSSP2 confocal microscope equipped with a CCD camera (CoolSnap HQ; Photometrics).

### RNA extraction

At the indicated time postincubation cells were collected and RNA was isolated by using the RNeasy Mini Kit (Qiagen, Valencia, CA, USA) following manufacturer’s recommendations. Total RNA was treated with DNA-se (Qiagen) and reverse transcribed using the Super Script III First Strand synthesis system (Invitrogen) for RT-PCR and Q-PCR assays.

### Genechip and data analysis from neurospheres

Total RNA extracted from transduced cells was tested on disposable RNA chips (Agilent RNA 6000 Nano LabChip kit) to determine the concentration and purity/integrity of RNA samples using Agilent 2100 bioanalyser. cDNA synthesis, biotin-labeled target synthesis, hybridization to HG-U133 plus 2.0 GeneChip (Affymetrix) arrays, staining and scanning were performed according to the standard protocol supplied by Affymetrix. For each probe set on each array, a detection call of Present, Absent or Marginal was made. Detection calls were made using the affy R/Bioconductor package [33]. Background corrected raw data were Log2-transformed and quantile-normalized following the Robust Multichip Average (RMA) procedure using R (Bioconductor) [34]. Differentially expressed genes were obtained with *limma* package [35], performing pairwise comparison between the mock and vector transduced hmNPCs and picking up probe sets showing a present call and a fold change > ± 1.5. A moderated t-test was performed between transduced and untreated groups selecting probe sets with a p-value ≤ 0.05. The data set containing the Affymetrix probe identifiers, selected as differentially expressed in transduced hmNPCs, and the corresponding fold changes, were uploaded into g:Profiler (updated version 23/7/2013). gGOSt Gene Group Functional Profiling was set with the following parameters: i) significant only, ii) hierarchical sorting, and iii) as specified in the results section either no filtering or hierarchical filtering best per parent group (strong). Heat maps of differentially expressed genes and belonging to selected enriched functions were constructed by using Excel 2007 (Microsoft Office package). Genes were categorized based on the annotations on g:Profiler [36]. The entire microarray data set was submitted to the Gene Expression Omnibus repository with the accession number GSE62687 (http://www.ncbi.nlm.nih.gov/geo/query/acc.cgi?acc=GSE62687).

Comparative analyses were performed on data reported here and data submitted to the Gene Expression Omnibus repository with the accession number GSE47130 applying the same statistical approach described above.

### PCR and Q-PCR

cDNAs from mock and treated hmNPCs were used for validation of selected genes. RT-PCR GFP expression was evaluated by using Platinum Blue PCR SuperMix (Life Technologies) using the following primers pairs For 5’-GCCGACCATTATCAACAGAACA-3’ and Rev 5’- TGGTTGTCTGGGAGGAGCAC-3’; for sample quantification the following beta-actin primers were used: For 5’-CGGCATCGTCACCAACTG-3’ and Rev 5’-ggcacacgcagctcattg-3’.

Q-PCR GFP and single gene expression was measured by TaqMan (Universal PCR Master Mix, Applied Biosystems), using the following TaqMan Gene Expression Assays (Applied Biosystems): GFP, For 5’- CAACAGCCACAACGTCTATATCATG -3’ and Rev 5’- ATGTTGTGGCGGATCTTGAAG-3’, EN1 batch ID Hs00154977_m1, EN2 batch ID Hs00171321_m1, CENPM batch ID Hs00894703_g1, PLK4 batch ID Hs00179514_m1, KIF14 batch ID Hs00208408_m1, BIRC5, batch ID Hs04194392_s1, FANCD2, batch ID Hs00945455_g1, MAD2L1, batch ID Hs01554513_g1, TUJ-1 batch ID Hs00909233_m1, and TH batch ID Hs00165941_m1, TLR3 batch ID Hs01551078_m1, CD44 batchID Hs01075861_m1, TLR4 batch ID Hs00152939_m1. Sample normalization was carried out on the basis of GAPDH expression (Applied Biosystems, TaqMan Gene Expression Assay, batch IDs Hs99999905_m1). GAPDH was unchanged in transduced as compared to mock samples. Reactions were performed using the Applied Biosystems PRISM 7300 Real Time PCR System. To obtain relative quantification with respect to the undifferentiated mock cells, quantification cycle values (Cq, [37]) were exported directly into an EXCEL worksheet for analysis, and the data were calculated with the 2^–ΔΔCq^ method [38].

### Statistical analyses

As indicated in the figure legends, data are reported as means ± standard deviation (SD) of triplicates or more data obtained from at least two independent experiments. Data were analyzed using two-tailed Student’s t-test.

## Results

### Transduction of neurospheres with viral vectors

**Fig 1:**
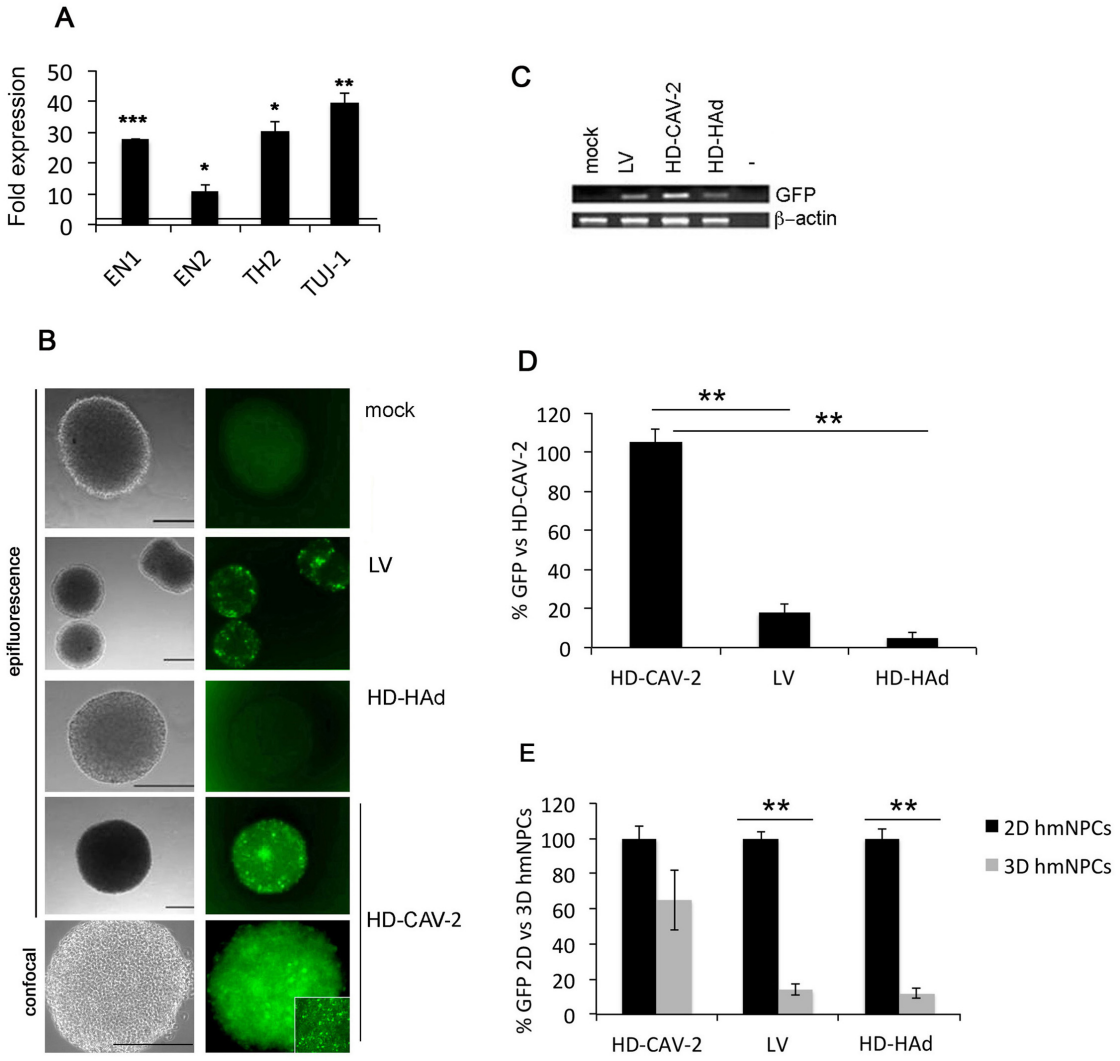
HD-CAV-2 efficiently transduces human neurospheres. Cultures of differentiated hmNPCs were transduced at an MOI of 1000 vg/cell with HD-HAd, LV, and HD-CAV-2, or mock treated and analyzed 5 days posttransduction. A) Single gene expression of dopaminergic markers quantified by Q-PCR in 3D undifferentiated and differentiated neurospheres. The horizontal line on the graphs corresponds to the average value measured in undifferentiated samples. Results are the average of two experiments performed in duplicate. p-values were calculated by Student t-test, * p<0.05; ** p<0.01; ***p<0.001. B) Representative images of transduced neurospheres. Cells were mounted in DAPI-Vectashield and analyzed with an epifluorescence microscope or confocal microscope as indicated. Scale bars, 200 μm. C) Semi-quantitative RT PCR on RNA extracted from transduced neurospheres. D) Q-PCR quantification of GFP expression performed on transduced neurospheres. Data are expressed as % of HD-CAV-2-samples GFP expression levels. E) Efficiency of transduction on 3D cultures quantified by Q-PCR as compared to 2D cells. Data are expressed as % of values obtained with the same vector on 2D cultures. D) and E) Results are the average of two experiments performed in duplicate. p-values were calculated by Student t-test, ** p<0.01.

As a first step for the analysis of the effect of HD-CAV-2 on human differentiated neurospheres, we monitored the expression of dopaminergic markers (Fig 1A). Compared to undifferentiated samples, differentiated neurospheres displayed significant and robust levels of the midbrain markers EN1 and EN2 and (30- and 10-fold increase, respectively) of the dopaminergic markers TH2 and TUJ-1 (30- and 40-fold increase, respectively). These data are in line with previous results applying the same differentiation and culture protocol [25,26,31].

Neurospheres were incubated with HD-CAV-2, LV and HD-HAd vectors. All vectors contained a GFP expression cassette and were used at the MOI of 1000 vg/cell. Five-days postincubation, HD-CAV-2-treated neurospheres displayed robust GFP expression as assessed by wide field and confocal fluorescence microscopy (Fig 1B). By contrast, HD-HAd and LV-treated neurospheres did not display notable GFP signals. Gene expression data was consistent with the fluorescence data on the efficacy of HD-CAV-2 transduction, and showed that, compared to LV and HD-HAd, HD-CAV-2 more efficiently transduced human neurospheres (Fig 1C and 1D). Indeed, the 3D neural cell model accentuated the difference in transduction efficiency of LV and of HD-HAd, but not for that of HD-CAV-2. The levels of HD-HAd and LV-mediated GFP expression were reduced by ~90% in differentiated neurospheres, as compared to 2D cultures of hmNPCs transduced using similar conditions. By contrast, the transduction efficacy was comparable for HD-CAV-2 (Fig 1E).

Taken together, these data indicate that HD-CAV-2 efficiently transduced human differentiated neurospheres, and showed that the 3D model was well suited for studying the molecular impact of gene transfer vector on human neural cells.

### Transcriptome analysis of HD-CAV-2 transduced neurospheres

**Fig 2:**
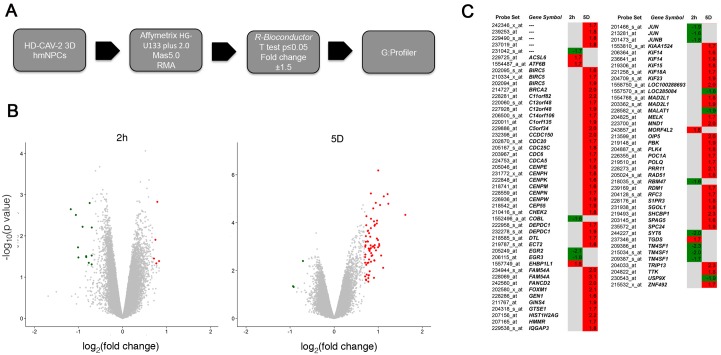
High throughput Genechip analysis of HD-CAV-2 neurospheres. A) Schematic representation of chip analysis workflow. B) Volcano plots: the gene expression difference between transduced samples and mock samples (fold change) is plotted on the x axis in log2 scale, and p-values are plotted on the y axis (–log10 scale). Upregulated and downregulated probes are indicated in red and green, respectively. All control probes were excluded from this analysis. Values represent the average of the three independent replica experiments. C) Probes modulated by HD-CAV-2 at 2h and 5 days posttransduction. The relative fold change values are indicated; in red, upregulated probes, in green, downregulated, and in grey, probes with unmodified expression with respect to mock.

To characterize the toxicogenomic signature and the biological properties of HD-CAV-2-neural cells interaction, human differentiated neurospheres, derived from the BNA hmNPC batch, were incubated with the vector at an MOI of 1000 vg/cell. RNA was extracted at 2 h and 5 days postincubation to analyze both early and mid-late events following vector effects. RNA was tested on HG-U133 plus 2.0 Affymetrix genechips, which included greater than 47,000 transcripts. Mas5.0 and RMA were used for raw data elaboration, R Bioconductor *limma* package was then applied for statistical evaluation of modified genes. Three independently transduced and mock samples, at the two time points, were tested. A p-value ≤0.05 and a fold change > ±1.5 were used as threshold values (Fig 2A). These parameters were chosen to identify statistically significant variations (p-value ≤0.05), and include small effects (fold change > ±1.5), that could be relevant to define the “full” picture of the potential molecular toxicity of HD-CAV-2 on human cells. The global impact of the vector was limited, indicating modest potential toxicity at this MOI. But a significant response was detected at 5 days postincubation (Fig 2B). Seventeen probes were modulated by HD-CAV-2 at 2 h postincubation (Fig 2C), 12 were downregulated and 5 upregulated. At 5 days, the modulation included a total of 72 probes, of which only 3 were downregulated and the rest induced (Fig 2C). At both time points, the extent of single probe modulation ranged from a fold-change of -2.3 to 3.1.

**Fig 3:**
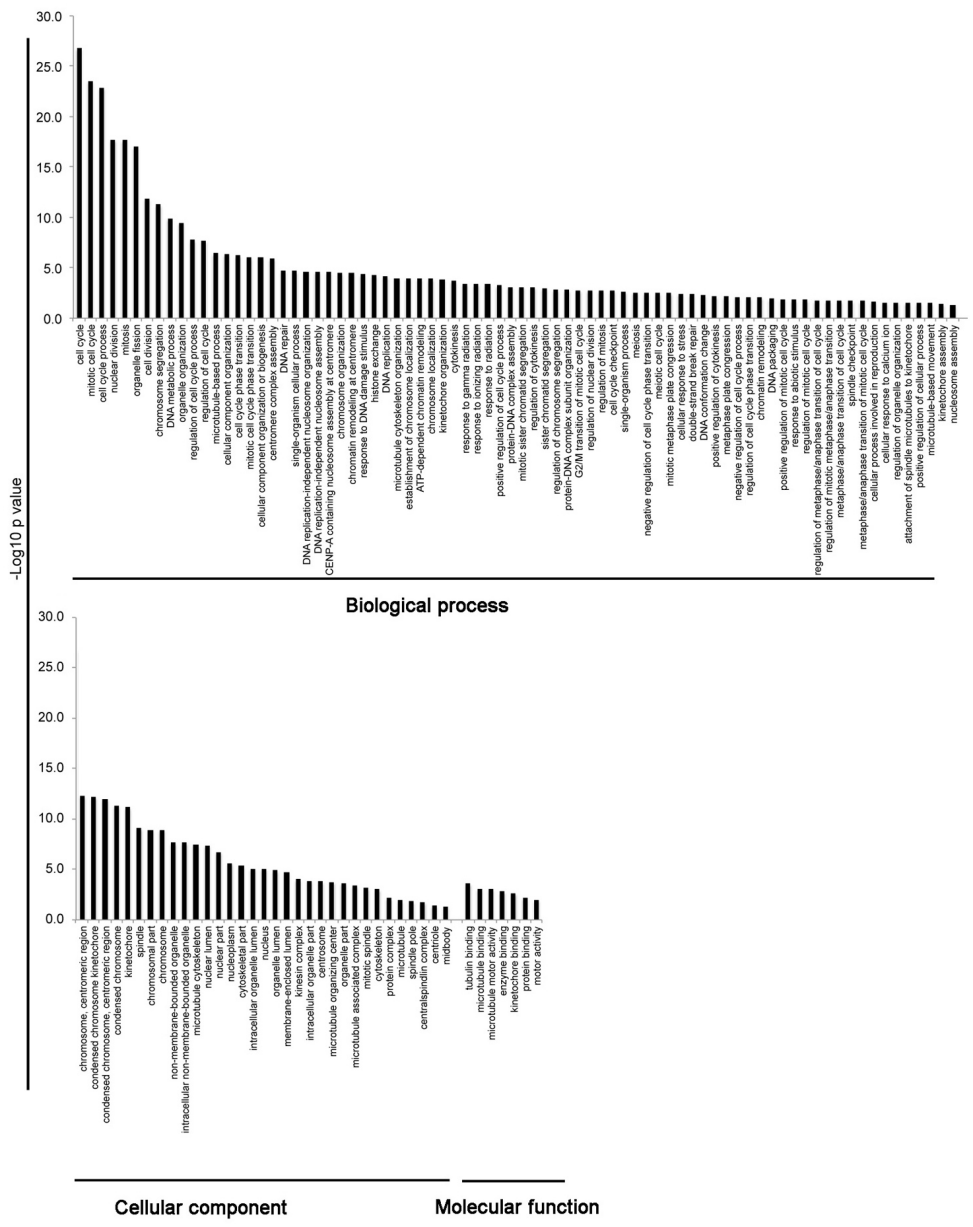
Biological processes, cellular components and molecular functions modulated by HD-CAV-2 in human neurospheres. The bioinformatic online software g:Profiler gGOSt set as significant only, hierarchical sorting and no hierarchical filtering was used to classify the genes transcriptionally modulated upon transduction of human neurospheres at both 2 h and 5 days time points. Among the biological processes the g:Profiler analysis identified cell cycle, mitosis, DNA damage, microtubule and centromere-related processes. The centromeric and microtubule aspects were revealed also by the cellular component category, as much as by the molecular function grouping. The full list of probes modulated in the groups is detailed on S1 Table.

**Fig 4:**
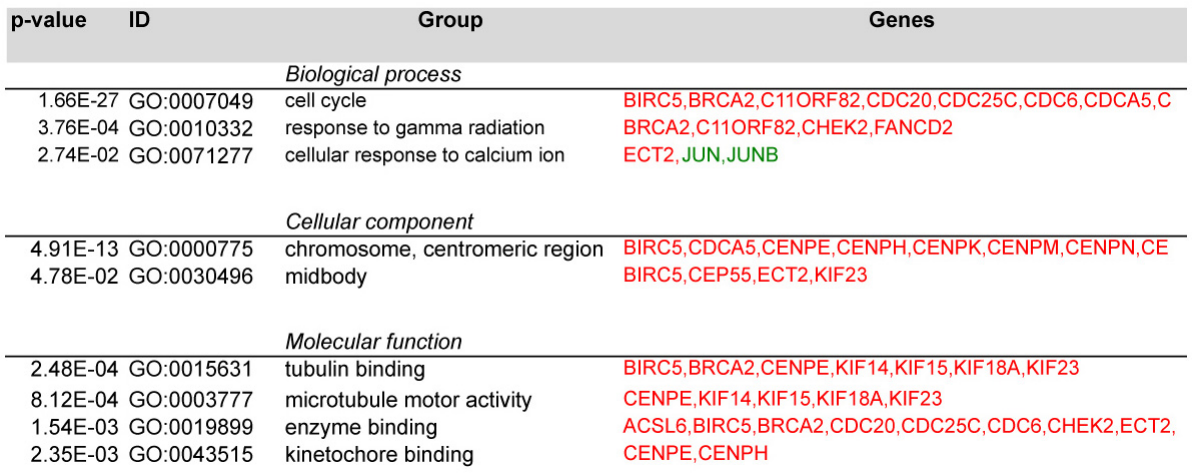
Single genes and relative groups identified through stringent g:Profiler analysis. g:Profiler gGOSt was set with the following parameters: i) significant only, ii) hierarchical sorting, and iii) hierarchical filtering best per parent group (strong) to identify most robustly gene groups regulated by HD-CAV-2 transduction. These include the cell cycle and DNA damage related processes, and the centromeric-related metabolism. Interestingly, several microtubule-associated molecular motors were part of the transcriptomic picture of HD-CAV-2-neurospheres. Specifically, infection with HD-CAV-2 transduction induced the modulation of multiple kinesins. In red, upregulated probes, in green, downregulated, and in grey, probes with unmodified expression with respect to mock.

To interpret the biological significance of global gene modulation, the full list of probes modulated by HD-CAV-2 was uploaded into the bioinformatic online software g:Profiler. By g:GOSt filtering we identified groups including biological processes, cellular components and molecular functions (Fig 3 and S1 Table). Among the biological processes in silico analysis identified cell cycle, mitosis, DNA damage, microtubule and centromere-related processes. The centromere and microtubule aspects were revealed also by the cellular component category, and by the molecular function grouping. To highlight single genes involved in these processes, g:GOSt was performed with greater stringency (i.e. g:GOST hierarchical filtering parameter: best per parent group, strong). Single genes and relative groups identified through this analysis are reported on Fig 4. The cell cycle and DNA damage-related processes modulated by HD-CAV-2 in neurospheres are defined by the transcriptional induction of genes including BIRC5, BRCA2 and FANCD2. On the other hand, the positive modulation of the centromeric genes CENPE, CENPH, CENPK, CENPM, CENPN, CENPW suggests that HD-CAV-2 induces the modulation of the centromeric-related processes. Interestingly, several microtubule-associated molecular motors were part of the transcriptomic picture of HD-CAV-2-neurospheres. Specifically, transduction with HD-CAV-2 induced at day 5 the modulation of the kinesins KIF14, KIF15, KIF18A and KIF23.

### Comparative analyses of the response to HD-CAV-2 of 2D and 3D cultures

**Fig 5:**
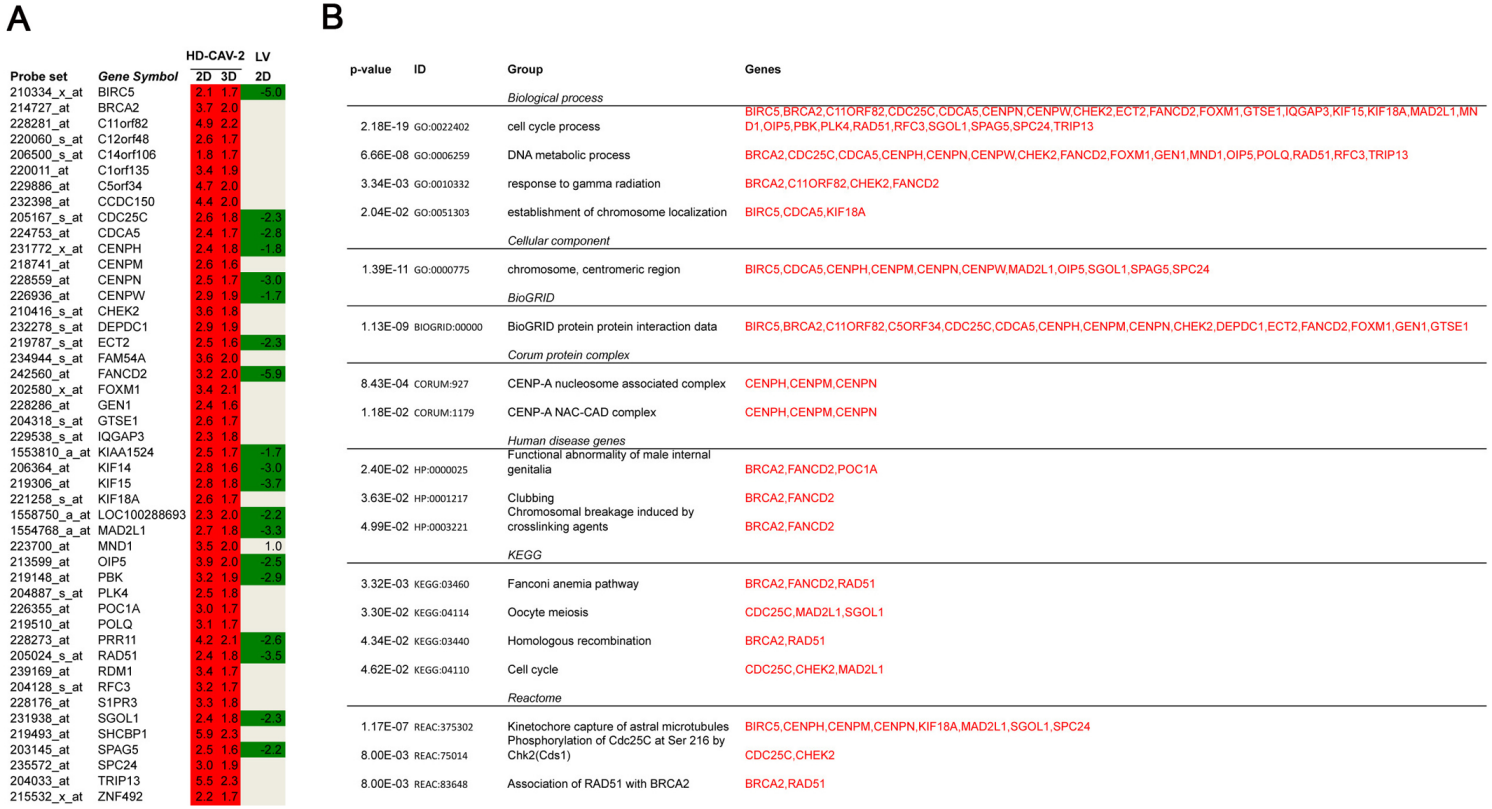
Comparative analysis of the response of 2D and 3D cultured hmNPCs to HD-CAV-2. In silico comparison of transcriptome data obtained from 2D hmNPCs transduced at an MOI of 1000 with HD-CAV-2 or with LV, with data obtained from HD-CAV-2 neurospheres. A) Comparative analysis revealed that 46 probes were found modulated in both 2D and 3D hmNPCs transduced with HD-CAV-2 and with the same sign, while a completely non-overlapping pattern is observed in LV-cells. B) g:Profiler gGOSt set as significant only, hierarchical sorting, and hierarchical filtering best per parent group (strong), was used to classify the genes commonly modulated in 2D and 3D culture conditions by HD-CAV-2. The analysis underlines the significant modulation of DNA damage response-, centromeric and microtubule metabolism-related probes. In red, upregulated probes, in green, downregulated, and in grey, probes with unmodified expression with respect to mock.

**Fig 6:**
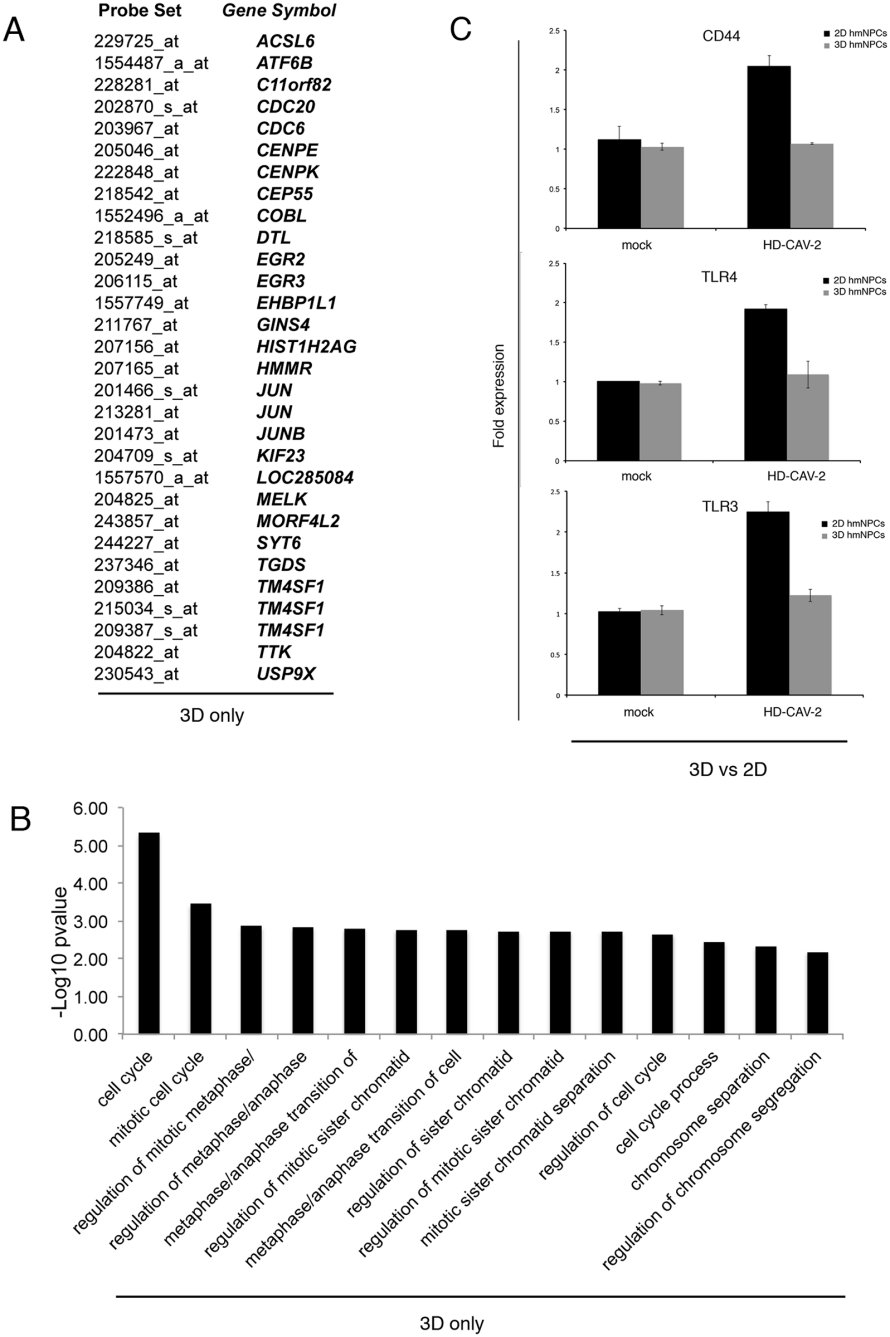
Probes regulated uniquely in 2D or 3D cultured hmNPCs by HD-CAV-2. A) and B) In silico comparison of transcriptome data obtained from 2D hmNPCs transduced at an MOI of 1000 with HD-CAV-2 with data obtained from HD-CAV-2 neurospheres. A) Comparative analysis revealed that 30 probes were found modulated in 3D hmNPCs transduced with HD-CAV-2 and not in 2D cultures. B) g:Profiler gGOSt was used to classify the probes uniquely modulated in 3D culture conditions by HD-CAV-2. The full list of probes modulated in the groups is detailed on S2 Table. C) Single gene expression was quantified by Q-PCR in differentiated 2D and 3D neural cells transduced with HD-CAV-2 and mock treated, at 5 days postincubation. Results are the average of two experiments performed in duplicate. The difference between mock and HD-CAV-2 treated 2D cells was significant with p<0.05 for all the analyzed genes and the difference between mock and HD-CAV-2 treated neurospheres was not statistically significant. p-values were calculated by Student t-test.

Transcriptome data related to the effect of LV and of HD-CAV-2 in 2D cultures of human neurons [22] (raw data are available at the GEO site http://www.ncbi.nlm.nih.gov/geo/query/acc.cgi?acc=GSE47130) was used to compare the 2D and neurosphere responses to HD-CAV-2. We found that 46 probes were modulated both in the 2D and 3D conditions (Fig 5). These results obtained in different cell batches (BNA and 3821 for 2D and BNA for 3D samples) and two different culture conditions (2D and 3D) strengthened the reliability of the analyses reported on Fig 2, and allowed us to identify the transcriptome alterations specifically related to human neural cells transduced by HD-CAV-2. The g:GOSt analysis of the 46 probes commonly modulated by HD-CAV-2 in the 2D and 3D neurospheres, highlighted cell cycle, DNA metabolism, the centromeric pathway, and the microtubule related processes as being the stringent transcriptional signature of HD-CAV-2 transduction. The comparison of HD-CAV-2 induced genes to the data obtained in the 2D system with LV reinforced the concept that the response of neural cells to HD-CAV-2 was vector specific. Indeed, out of the 46 probes modified by HD-CAV-2 in differentiated hmNPCs, 20 were modulated by LV with opposite sign and the rest were unchanged. We then compared the responses to HD-CAV-2 in 2D and 3D cultures to identify probes uniquely modulated in 3D cells as compared to 2D. This analysis showed that 30 probes were specific to the neurosphere response to HD-CAV-2 (Fig 6A); g:GOSt analysis on this probe list confirmed that the cell cycle, mitotic/centromeric related gene groups are part of the 3D related signature of HD-CAV-2 transduction (Fig 6B and S2 Table). We then asked whether genes that were uniquely regulated in 2D (S3 Table) could have been missed in the chip analysis of the neurospheres. We then selected three genes, TLR3, TLR4 and CD44, among those that were found modulated only in 2D samples belonging to innate immune response gene group that was not found modulated in the neurosphere samples and analyzed them by Q-PCR in both 2D and 3D samples incubated with HD-CAV-2. Q-PCR data confirmed that their expression was upregulated in 2D hmNPCs and unchanged in neurospheres (Fig 6C). These results taken together suggest both common and culturing condition-specific aspects of the response of differentiated hmNCPs to HD-CAV-2.

### FK^CAV^ and HD-CAV-2 exert specific effects on human neurons

**Fig 7:**
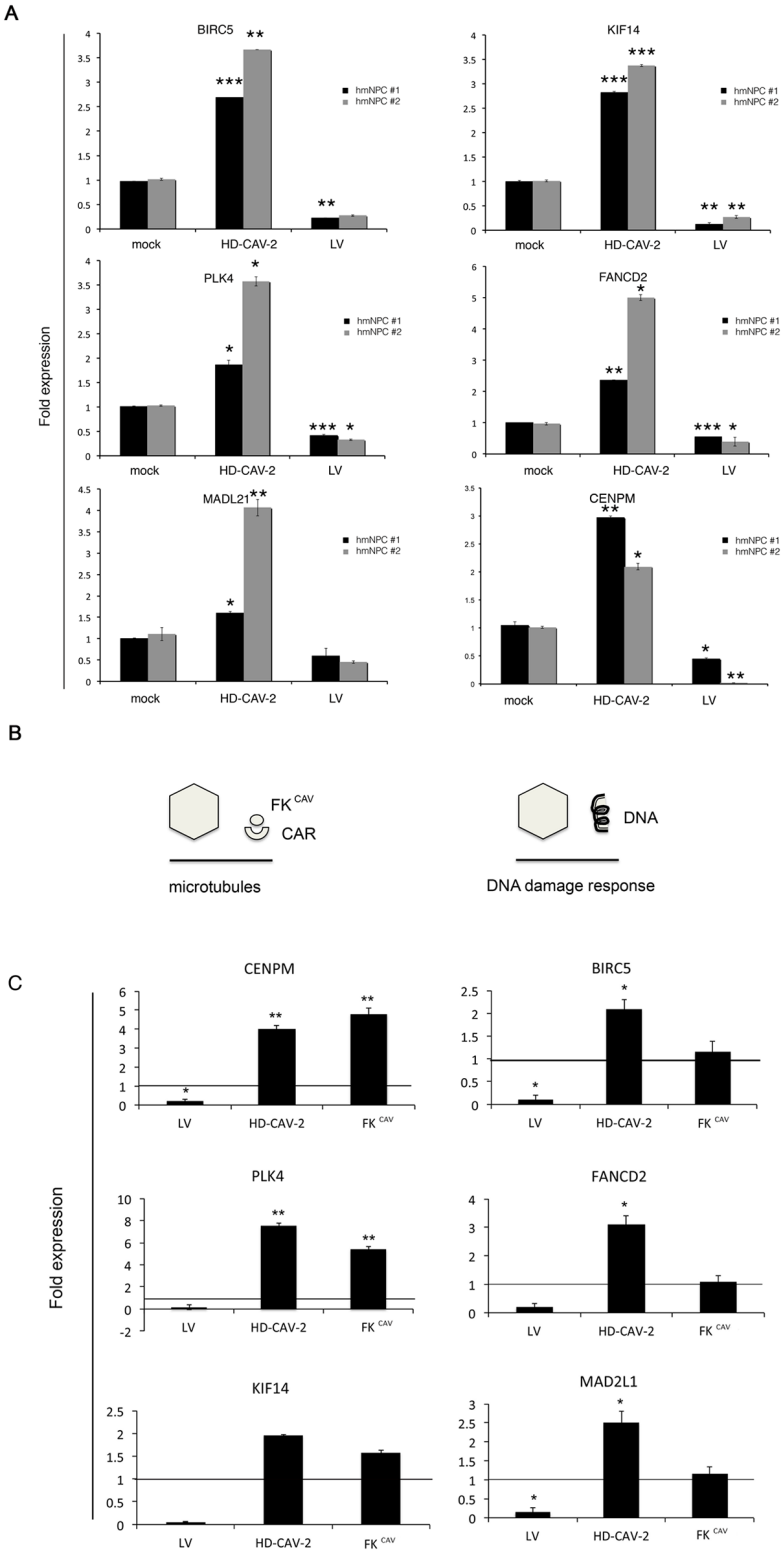
HD-CAV-2 and fibre knob modulate genes related to microtubule metabolism. A) Single gene expression validation by Q-PCR in differentiated neural cells derived from two independent hmNPC batches (#1, BNA and #2, 3821) transduced with HD-CAV-2, LV, mock treated, at 5 days posttransduction. Results are the average of two experiments performed in duplicate. For each gene for the difference between mock and HD-CAV-2 treated cells and between mock and LV treated cells p-values were calculated by Student t-test, * p<0.05, ** p<0.01, *** p<0.001. B) Schematic representation of the experimental hypothesis suggesting that HD-CAV-2 and fibre are able to modulate microtubule related genes, possibly through an interaction with the CAR receptor. C) Single gene expression was quantified by Q-PCR in differentiated neural cells transduced with HD-CAV-2, LV, mock treated, or incubated with purified fibre knob (FK^CAV^) at 5 days posttreatment. The horizontal line on the graphs corresponds to the average value measured in mock samples. Results are the average of two experiments performed in duplicate. p-values were calculated by Student t-test, * p<0.05; ** p<0.01.

In our previous study detailing the effect of viral vectors on 2D differentiated hmNPCs we hypothesized that the modulation of the DNA damage response was linked to vector genomes, and that in the absence of virus-activated counteracting functions, can activate a DNA damage pathway [22]. This response is particularly characteristic for a linear, double-stranded DNA genome of adenoviruses where the ends are rapidly recognized as damaged DNA. Here we add to this interpretation, the hypothesis that the HD-CAV-2 induced modulation of microtubule-related genes observed in human neural cells could be linked to the engagement of CAR, through FK^CAV^ binding. To test this hypothesis we selected 6 genes among those positively and selectively modulated by HD-CAV-2, 3 related to DNA metabolism (BIRC5, FANCD2, MAD2L1), and three to the centromere and microtubule metabolism (CENPM, KIF14, PLK4). We firstly validated their modulation in two independent hmNPCs batches by Q-PCR (Fig 7A). We then probed their modulation in response not only to HD-CAV-2 and LV but also to FK^CAV^, which, when incubated with neurons, co-localizes and is internalized with CAR [11,32]. In line with our hypothesis (Fig 7B), the DNA metabolism-related genes were positively regulated by HD-CAV-2 but not by FK^CAV^, and centromere and microtubule related genes were upregulated in response to both HD-CAV-2 and FK^CAV^ (Fig 7C). LV showed a fully different biological effect, reinforcing the hypothesis that the modulation of these pathways is vector specific.

## Discussion

Viral vectors including AAVs, LVs and Ads have been suggested for brain gene therapy because they can transduce human neurons with varying efficacy [1,2]. HD-CAV-2 vectors have properties that make them particularly suited for gene transfer to the neurons, including the paucity of pre-existing humoral immunity in humans, efficient transduction, and long-range axonal trafficking [3,39]. These characteristics have been related to the HD-CAV-2 attachment molecule, CAR. Detailed investigations have demonstrated a direct link between FK^CAV^ binding and CAR trafficking in the neuron [10,11]. These observations prompted us to analyze the transcriptional response induced by HD-CAV-2 and human neural cells.

Studies addressing vector transduction have been mainly performed in vivo in nonhuman models or in human 2D cultures. In a 2D model of dopaminergic differentiated hmNPCs we assessed the impact of HD-CAV-2 on the human neural cells [22]. Here, we tested HD-CAV-2 vectors in a 3D model that better mimics the architecture of cell-cell and cell-extracellular matrix interactions in the human brain. In 3D cultures of differentiated hmNPCs, the cells connect in a complex tissue-like structure, eliciting spontaneous Ca^2+^ transients, voltage- and glutamate-dependent currents, synaptic vesicle trafficking and release of dopamine in response to stimuli [26]. During the differentiation process neurospheres ultrastructure is subject to intense remodeling through complex arborization of the cellular network and undergoing synaptogenesis and generation of dendritic spines. The analysis of the impact of HD-CAV-2 in this system is particularly pertinent due to these intrinsic properties of the neurospheres.

The first observation that distinguished differentiated neurospheres from 2D cultures was that the 3D cultures accentuated the difference in transduction efficacy. These results reflect the efficient and preferential transduction of neurons by CAV-2 vectors in the brain parenchyma in vivo [3]. Incubation of human neurospheres with CAV-2 vectors allows transgene expression in the inner part of the differentiated neurosphere [31]. It is probable that the entry pathway of CAV-2, which exploits CAR functions, contributes to efficient trafficking in the neurosphere and consequent high transduction levels. Conversely, HD-HAd and LV exploit different and complementary entry pathways. HD-HAd binds CAR, but it can also bind other cell surface molecules that likely lead to unproductive transduction pathways. LV vector is VSV-G pseudotyped, and a recent study described the LDL receptor as the major entry port of VSV-G-pseudotyped vectors in mammalian cells [40].

We chose two time points, 2 h and 5 days postincubation, to identify early and mid-late events following vector addition to neuroshperes. We observed that at 2 h the response was quantitatively limited. Conversely, at 5 days the modulation of the neurosphere transcriptome included a larger number of significantly modulated probes. Notably, the 5 days time point is expected to reflect the events accumulating in the cells starting from vector addition, because neurons cell division is mostly arrested in these differentiated 3D cultures. Stringent statistics on the modulated genes highlighted three main traits in the transcriptome response of the neurospheres to HD-CAV-2: the regulation of cell cycle and DNA metabolism, centromeric and microtubule related probes.

Comparing data obtained in 3D samples to those previously described for 2D cultured hmNPCs [22] indicated that DNA metabolism-related gene modulation is a net marker of HD-CAV-2 mediated transduction, in both 2D cultures of hmNPCs and neurospheres. The microtubule and centromeric probes were also modulated both in 2D and 3D conditions, although in neurospheres the modulation of these gene groups was more clear-cut. One aspect that distinguished the 2D and 3D samples was that of the innate response, present in 2D samples and not in neurospheres [22], possibly as a consequence of the specific neuronal differentiation properties of the neurosphere cells as compared to 2D cultured hmNPCs [31].

The connection between the adenoviral DNA and the activation of the DNA damage pathway was anticipated because adenoviruses encode specific functions to counteract the activation of the DNA damage pathway. The viral E4orf3 protein can block the activity of the Mre11-Rad50-NBS1 DNA repair complex. The alteration of the DNA damage response pathway is important for the virus life cycle because it blocks detrimental aspects of checkpoint signaling during virus infection and inhibits concatemerization of viral DNA molecules [41,42]. Here we detected the modulation of DNA metabolism genes, including BIRC5, BRCA1, FANCD2 and RAD51. We showed that three genes selected among those implicated in DNA metabolism, BIRC5, FANCD2 and MAD2L1 were upregulated by HD-CAV-2, while their expression was not altered when only FK^CAV^ was added to the cells. These results taken together support the hypothesis that HD-CAV-2 infection induces a DNA damage response, an aspect that should be taken into account, especially when administrating the vectors in humans, at high doses, in sensitive organs.

The modulation of microtubule and centromeric probes is an interesting characteristic of the transcriptome response to HD-CAV-2. The regulation of single probes belonging to these functional groups was represented in the 2D model system, but in the neurosphere model it is a quantitatively dominant trait of the response. The centromere is a specialized chromatin domain acting as a substrate for the assembly of the kinetochore during mitosis, which mediates chromosome segregation during cell division. CENPA, is the centromere specific histone H3 variant. CENPH, N and M have been co-isolated with CENPA and are found in the CENPA nucleosome [43]. CENPK is a CENPA nucleosome distal component. The kinetochore interacts with microtubules of the mitotic spindle and is linked to microtubule dynamics [44]. CENPs modulation by HD-CAV-2 was, in our system, detected in parallel with that of microtubules-related probes. Indeed, we found that numerous kinesins, the microtubule motors, were transcriptionally activated by HD-CAV-2. We also observed the modulation of PLK4, a Polo-like kinase family member, important in mediating microtubule nucleation [45].

These results suggest that the microtubule network could be dynamically altered by HD-CAV-2.

A possible explanation for the modulation of CENPs could be linked to the presence of viral DNA. Zeitlin and co-workers showed that double-strand DNA breaks recruit CENPA, along with CENPN, CENPT and CENPU [46]. Another explanation could be the link between HD-CAV-2, CAR and microtubule dynamics. To address this question, we tested the modulation of CENPM, PLK4 and KIF14, in response to HD-CAV-2 and to FK^CAV^. We found that both agents modulated these probes. These results, although restricted to a limited number of genes, suggested that CAR engagement and possibly release of homodimeric CAR interactions [11] could be responsible for the complex alteration of microtubule and related kinetochore probes.

Together the data presented in this work suggest that HD-CAV-2 impacts human neural cells transcriptome via the vector genome DNA and possibly via the FK interaction with CAR. Future investigations on the biochemistry of biological processes highlighted by probe modulations will help to unravel whether they are operative in HD-CAV-2 transduced cells and/or whether particular signaling cascades are functionally engaged upon CAR binding, and could be taken into consideration when designing gene therapy experiments based on viral vectors. Finally, these results underline the importance of using updated brain related model systems to make specific properties of viral vectors to emerge.

## Supporting Information

S1 Table Gene groups identified through g:Profiler analysis. The bioinformatic online software g:Profiler gGOSt set as significant only, hierarchical sorting, and no hierarchical filtering was used to classify the genes transcriptionally modulated upon transduction of human neurospheres at both 2h and 5 days time points. T indicates the number of genes associated in functional terms, Q is the number of genes in input list.
(XLSX)

S2 Table Functional classification of probes modulated uniquely in 3D hmNPCs by HD-CAV-2. g:Profiler gGOSt was used to classify the probes uniquely modulated in 3D culture conditions by HD-CAV-2. T indicates the number of genes associated to functional term, Q is the number of genes in input list.
(XLSX)

S3 Table List of probes modulated uniquely in 2D hmNPCs by HD-CAV-2. In silico comparison of transcriptome data obtained from 2D hmNPCs transduced at an MOI of 1000 with HD-CAV-2 with data obtained from HD-CAV-2 neurospheres.
(XLSX)
